# 

**Published:** 1832

**Authors:** 


### INDEX.

A.

Absorption, Pulmonary Experiments on, 121.
of the Iris, 101.
Abernethy's Lectures on Surgery, 274.
Biography of, 323.
Abdominal Peessure in Parturition, 128.
Abortion, Culpable, 488.
Abortives, supposed class of, 648.
Abstinence, Extraordinary, 637.
Acetate of Morphia, 130. 635.
Acid, Hydrocyanic in vomiting, 150.
Iodic, a test for Vegito Alkalis, 499.
Acetic, Poisoning by, 633.
Acetabulum, Fracture of, 292.
Acupuncturation of the Arteries, 502.
Anatomy, Massachusetts Bill concerning, 166.
Aneurism of the Axillary Artery cured by tying the Subclavian, 651.
Analysis of the Sulpher Springs at Nashville, Tenn. 648.
Arnott's Elements of Physics.397.
Ascarides, Fatuity produced by, 477.
Atmospheric Electricity, Influence of on the Eye, 651.

B.

Bones, Inflammation of the medullary Tissue of, 637.

C.

Cantharadin, Oil of, 650.
Catechism of Health, Review of, 100.
Cholera, 449. 593.
Infantum, 530.
Charcoal, Spontaneous Combustion of, 498.
Constitutional disease produced by local Irritation, 313.
Consumption, cases of, 487.
Influence of occupation in the developement of, 499.
Compression, Inflamation of the Saphena vein cured by, 127.
Colchicum Autumnal,
in Rheumatism, 335.
Cyanuret of Potassium successful in Neuralgia, 630.

D.

Davy compared with Wollaston, 165.
Dislocation of the thigh bone, cases of, 153.
of the Sacro Iliac Syniphlsis, 297
Dropsy in Pregnancy, 497.
Duodenum, ulceration of, produced by a Spoon, 479.
Dyspepsia, causes of, 566.

E.

Education, Medical, Drake's Essays on, 9. 179. 505.
Emetics, Drakes observations on, 543.
Elaterium, Active principle of 291.

Epilepsey cured by the Trephine, 646.
Excoriation of the Mamma, 631.
Fœtus in Utero, Small Pox communicated to, 142.
Fracture of the Acetabulum, 292.
of the Scapula, 295.
Fumigation Sulphurous, 144.
G.
Gall Bladder, Congenital absence of, 316.
Gaseous Tumors of the Uterus, 141
Generation of Steam by heated metals, 498.
H.
Harelip, Remarks on the operation for, 129.
Hernia, Radical cure of, 151.
New Method of Treating 152.
Heart, wound of, 161.
Energy of its contractions a guide in venesection, 305.
Health, Journal of, 199.
Hemorrhage from extraction of teeth, 478.
Hemiplegia, Intermittent, 474.
Hooping Cough, treatment of, 307.
Human voice, 470.
Hydrophobia, Drakes case of, 100.
I J.
Injections, description of an Apparatus for their administration, 156.
Jaundice, Pathology of, 319.
Imbecility, Mental from the injury of a nerve, 493.
Infanticide, trial for, 280.
Iris, absorption of, 305.
Irritation, spinal, 258t 354, 462.
L.
Lectures, Abernethy's, 274.
Lawrence's, 423, 565.
Leeches poisoned by the blood of a patient, 324.
Lithotomy, 502.
Recto Vesical, 494.
Staughton's history of, 67.
Lithotrity, 478, 501.
M.
Magnesium, 513.
Mamma, Excoriation of, 631.
Medical Men, Responsibility of in a court of justice, 289.
Medical Education, Drake's Essays on, 9, 169, 500.
Statistics, 162, 286.
Medicine, Mitchell on the morality of, 23.
Meat of diseased animals; is it unwholeseme? 643.
Medullary Tissue of the long bones, Inflammation, 637.
Medico Chirurgical Transactions, 233.
Medical School of Paris, 617.
Medical Jurisprudence, 481, 488.
Mercury in Syphilis, 36.
Mental imbecility from the injury of a nerve, 493.
Morphia, Acetate of, 150, 635.
Muriate of, 291.
Muscles, Permanet involuntary contraction of, 303.
N.
Natural Philosophy, Arnott's, 211 397.
Nervous System, Flourens researches on, 177.
New remedy, 501.
Neuralgia treated by Cyanuret of Potassium, 630.
Neuralgic diseases, Teale on, 258.
O.
Obstetrics, Abdominal pressure in 128.
Oil of Cantharadin, 636.
Turpentine, 502.
Opium, 631, 502.
effects of eating, 627.
P.
Pharmacopœia of the United States, 119.
Pathology of Jaundice, 319.
Penis, Muscles for compressing the dorsal vein of, 316.
Pericardium and heart, wound of, 161.
Pessary, 147.

Phosphate of Quinine, 504.
Phthisis Pulmonalis, 121. 467.
Influence of occupation upon, 499.
Physiology, Lawrence's Lectures 498.
Piperine, 635.
Physics, Elements of, 207, 397.
Poisoning by Acetic Acid,
by bite of a Rattle snake, 480.
Posture, effect of upon the pulse, 136.
Potassium, Cyanuret of in Neuralgia, 480.
Polypus, 503.
Ptosis, new operation for, 305.
Pregnancy in Dropsy, 497.
new symptoms of, 162.
can a woman pass thro' this period without being conscious of it, 284.
Pulmonary absorption and exhalation, 121.
Putrefactive disorganization of the lungs, 140.

Q.

Quinine, Phosphate of, 504.

R.

Recto-vesical Lithotomy, 491.
Resuscitation, 494.
Retina, Increased sensibility of, 631.

S.

Saphena vein, Phlebitis cured by compression of, 127.
Sacro Iliac Symphysis, dislocation, 295.
Sarsaparilla comp. syrup of, 138.
Sarcomatous Tumor, Hitts case of, 559.
Testicle, 86.
Sciatic nerve, section of, 160.
Selerium, 4, 99.
Stammering, canse of, 132.
Statistics, Medical, 162, 286.
Skeleton, Living, 319.
Snake bite cured by cupping, 487.
Small Pox, Identity of with Cow Pox, 650.
communicated to Fœtus in Utero, 142.
Spinal Irritation, 258, 354, 462.
Steam, generation of by heated metals, 498.
Subclavian artery tied for axillary aneurism, 642.
Sulphur Springs at Nashville, Analysis of, 648.
Sulphureous Fumigations, 144.
Surgery, Abernethy's Lectures on, 274.
Syhilis, Staughton on the use of Mercury in, 36.

T.

Taliacotion operation, 129.
for restoration of the under lip, 317.
Tetanus caused dy a brass wire under the skin, 328.
Caused by inflamation of the spinal cord, 641.
Throat, wounds of, 298.
Trachetomy, 300.
Trephine in Fpilepsy, 646.
Twisting of the arteries, 501.
Tympanitic state of the Pericardium, 496.
Tympanium, perforation of, 297.
Typhus Icterodes, Dodd's observations on, 44.

U.

Uterus, Gaseous tumors of, 141.
Under Lip, Restoration of, 317.

V.

Vagitus Uterinus, 161.
Veratria, 321.
Vegetable Life, tenacity of, 650.
Vaccine Pustule, mode of inducing in the cow, 650.
Vomiting cured by Hydrocianic Acid, 150.
Voice, 470.
Voltaic Action, 497.

W.

Wollaston compared with Davy, 165.

Z.

Zoology, Lawrence's Lectures 423, 525.

668

### ANNUAL CATALOGUE
OF THE
### MEDICAL COLLEGE OF OHIO.
### SESSION 1831-2.
CINCINNATI.

---

### SUMMARY.

| | | |
|---|---|---|
| I. | Ohio, | 60 |
| II. | Kentucky, | 32 |
| III. | Virginia, | 13 |
| IV. | Mississippi, | 8 |
| V. | S. Carolina, | 2 |
| VI. | Indiana, | 5 |
| VII. | Pennsylvania, | 6 |
| VIII. | Louisiana, | 4 |
| IX. | Missouri, | 3 |
| X. | Tennessee, | 11 |
| XI. | Michigan, | 1 |
| XII. | Illinois, | 2 |
| XIII. | New York, | 1 |
| XIV. | Maryland, | 1 |
| XV. | Bermuda, | 1 |
| | | 150 |

---

The Annual Commencement for conferring the degree of Doctor of Medicine, was held on the 7th of March, 1832. The following are the names of the individuals on whom the Degree was conferrred.

| | |
|---|---|
| Prior G. Fore, | Kentucky. |
| Isaac T. Teller, | Ohio. |
| Moses M'Clintic, | Virginia. |
| William R. Falconer, | Louisiana. |
| James Hood, | Ohio. |
| Leonard Randal, | Tennessee. |
| P. W. Martin, | do. |

| | |
|---|---|
| R. T. Peyton, - - | Virginia. |
| Hiram Cox, - - | Ohio. |
| J. G. W. Mott, - - - | Louisiana. |
| J. P. Andrew, - - - | Ohio. |
| Jos. C. Carter, - - | Kentucky. |
| J. M. Duke, - - | Do. |
| James Lorimer, - - | Ohio. |
| Calvin Lea, - - - | Mississippi. |
| Jos. S. Hornsby, - - | Kentucky. |
| Chas. B. New, - - | Do. |
| R. B. Winlock, - - | Do. |
| Geo. W. Branch, - - | New York. |
| Chas. S. Tulley, - - | Indiana. |
| Edward Dawes, - - | Ohio. |
| Henry Craig, - - | Kentucky. |
| William Steele, - - | Ohio. |
| A. M. Newman, - - | Virginia. |
| Harvey Lewis, - - | Kentucky. |
| Wm. B. Towles, - - | Virginia. |
| W. L. Chamblin, - - | South Carolina. |
| J. W. Bigelow, - - | Ohio. |
| B. Easton, - - - | Indiana. |
| Richard Eberle, - - | Pennsylvania. |
| Hiel Morrison, - - | Ohio. |
| Ed. De Loughery, - - | Maryland. |
| James Hough, - - | Virginia. |
| Wm. Edwards, - - | South Carolina. |
| Adam Dickey, - - | Ohio. |
| John T. Shotwell, - - | Kentucky. |
| Total 36. | |

---

The Medical Faculty is as follows:—

JEDEDIAH COBB, M. D., *Professor of Anatomy and Physiology.*

THOMAS D. MITCHELL, M. D., *Professor of Chemistry and Parmacy*

JAMES M. STAUGHTON, M. D., *Professor of Surgery.*

CHARLES E. PIERSON, M. D. *Professor of Materia Medica.*

JOHN MOORHEAD, M. D., *Professor of Obstetrics and Diseases of Women and Children.*

JOHN EBERLE, M. D., *Professor of Theory and Practice of Medicine.*

### UNIVERSITY OF PENNSYLVANIA.

The number of students in this institution during the last winter, was eight hundred and forty-nine, of whom three hundred and ninety were attending the Medical Lectures. At a medical commencement, held March 29th, 1832, the Degree of Doctor of Medicine was conferred upon 136 gentlemen.

### UNIVERSITY OF MARYLAND.

At a public commencement held in the University on the 19th March, 1832, the Degree of Dr. of Medicine was conferred upon 61 gentlemen.

### TRANSYLVANIA UNIVERSITY.

There were attending the Lectures of this institution, during the past winter, 213 Medical students.

### THE WESTERN JOURNAL OF THE MEDICAL AND PHYSICAL SCIENCES.

Edited by DANIEL DRAKE, M. D. AND JAMES C. FINLEY, M. D.

The publisher of this Journal, the oldest of the kind in the western country, with peculiar pleasure informs his subscribers, that the increased patronage of the Profession will enable him to present the succeeding volume in an improved form, and to make such arrangements as will add greatly to its intrinsic value.

The senior Editor has engaged to devote to it his undivided leisure, with a determination to impart to it the character which the public have a right to expect from a work presented under the sanction of his name; and the junior Editor, having received assurances of regular and permanent assistance from a number of the most accomplished physicians of the west, will be enabled to assist him in imparting to its pages an interest and variety, in some degree, commensurate with the increasing expectations of the profession.

In addition to these arrangements, the publisher hopes by the incentives of a wide circulation, and of a liberal pecuniary compensation, to induce a general co-operation on the part of our subscribers, to furnish such cases of novelty and interest as so extensive a region must at all times afford.

Subscribers have a ready perception of the responsibilities of Editors, but they are less sensitive to the reciprocal obligation which rests upon them to supply the materials which impart value and interest to periodical publications.

The public have not been ignorant of the existence of causes which have hitherto retarded the progress of medical improvement in this country, but, we hope, the time has now arrived when the Profession, disencumbered of adverse influences, will co-operate harmoniously in establishing an independent literature which, by eliciting tbe talents, and adapting itself to the circumstances of the country, will possess irresistible claims upon their patronage.

Communications to be directed, free of expense, to the publisher. Reports of cases, &c. on subjects of general interest to the Profession, will be liberally and promptly paid for.

